# Highlights of the mini-symposium on extracellular vesicles in inter-organismal communication, held in Munich, Germany, August 2018

**DOI:** 10.1080/20013078.2019.1590116

**Published:** 2019-03-18

**Authors:** E. Bielska, P.R.J. Birch, A.H. Buck, C. Abreu-Goodger, R.W. Innes, H. Jin, M.W. Pfaffl, S. Robatzek, N. Regev-Rudzki, C. Tisserant, S. Wang, A. Weiberg

**Affiliations:** a Institute of Microbiology and Infection, School of Biosciences, University of Birmingham, Birmingham, UK; b Division of Plant Sciences, School of Life Science, University of Dundee (at James Hutton Institute), Dundee, UK; c Institute of Immunology and Infection Research and Centre for Immunity, Infection & Evolution, School of Biological Sciences, University of Edinburgh, Edinburgh, UK; d Centro de Investigación y Estudios Avanzados del IPN (Cinvestav), Unidad de Genómica Avanzada (Langebio), Guanajuato, Mexico; e Department of Biology, Indiana University, Bloomington, IN, USA; f Department of Microbiology and Plant Pathology, Center for Plant Cell Biology, Institute for Integrative Genome Biology, University of California, Riverside, CA, USA; g Division of Animal Physiology and Immunology, TUM School of Life Sciences, Weihenstephan, Technical University of Munich, Freising, Germany; h Biocenter, Ludwig-Maximilians University Munich, Martinsried, Germany; i Faculty of Biochemistry, Department of Biomolecular Sciences, Weizmann Institute of Science, Rehovot, Israel

**Keywords:** Extracellular vesicles (EVs), inter-organismal interactions, cell-to-cell communication, small RNAs, plants, nematodes, protists, fungi, oomycetes, bacteria

## Abstract

All living organisms secrete molecules for intercellular communication. Recent research has revealed that extracellular vesicles (EVs) play an important role in inter-organismal cell-to-cell communication by transporting diverse messenger molecules, including RNA, DNA, lipids and proteins. These discoveries have raised fundamental questions regarding EV biology. How are EVs biosynthesized and loaded with messenger/cargo molecules? How are EVs secreted into the extracellular matrix? What are the EV uptake mechanisms of recipient cells? As EVs are produced by all kind of organisms, from unicellular bacteria and protists, filamentous fungi and oomycetes, to complex multicellular life forms such as plants and animals, basic research in diverse model systems is urgently needed to shed light on the multifaceted biology of EVs and their role in inter-organismal communications. To help catalyse progress in this emerging field, a mini-symposium was held in Munich, Germany in August 2018. This report highlights recent progress and major questions being pursued across a very diverse group of model systems, all united by the question of how EVs contribute to inter-organismal communication.

Extracellular vesicles (EVs) have attracted growing attention due to their pivotal role in cell-to-cell communications and host-pathogen interactions. Despite the fact that EVs are found in both prokaryotes and eukaryotes and from unicellular to multicellular organisms, most studies to date have been motivated by potential applications in medicine, with a major focus on the development of EV biomarkers in humans as diagnostic and therapeutic tools for pathological diseases in the clinic. Research focussing on basic questions in EV biology, such as their cargo, biogenesis, secretion and cellular uptake, has been mostly restricted to mammalian systems, like humans and mice. Collecting more comprehensive data from whole-organism studies is challenging and is basically missing from non-animal species. Only a very few studies have focused on investigating EVs in non-mammalian systems such as bacterial, fungal, oomycete, invertebrate and plant species. The potential role of EVs in cross-kingdom and inter-organismal interactions in these systems is of particular interest.

Here, we report on the mini-symposium on EVs in inter-organismal communication held on the 30–31 August 2018 at the Ludwig-Maximilians University (LMU) in Munich, Germany, a continuation to the Sao Paulo ISEV workshop on EVs in cross-kingdom communication held in 2016 [1]. Both scientific meetings had common topics, but also exhibited their special characters and outcomes. At the ISEV workshop in Sao Paulo, one focus was laid on pathogen-derived EVs and the interaction with the host organism. A second key topic was put on the multitude of EV purification methods from pathogens to host matrices. A third point of discussion was the role of EVs in infectious diseases that are caused by pathogens from various kingdoms, e.g. virus, bacteria, fungi, protozoa and helminths. The mini-symposium on EVs in inter-organismal communication in Munich included additional and new aspects. Besides discussions on bacterial, fungal, oomycete and animal parasite EVs in cross-kingdom interactions, two new topics were implemented: first, plant EVs as carriers of sRNA and tiny RNAs, and second, the implementation of the MISEV guidelines in inter-organismal EV research. The Munich symposium brought together 55 participants from six different countries to communicate common interests, current opinions, and future directions in terms of EVs playing role in inter-organismal studies. A common goal of the researchers at the symposium was to understand the basic mechanisms underlying how EVs mediate inter-organismal communication. The meeting attendees suggested several new suitable model systems and proposed potential synergies and collaborations to enhance both the quality and quantity of EV research. Here, we present the most relevant outcomes and research topics introduced by the speakers at the mini-symposium on EVs in inter-organismal communications.

## EVs of plants in antimicrobial defence

Compared to work on mammalian EVs, work on plant EVs is in its infancy. Regente and colleagues described recovery of putative EVs from intercellular fluids of imbibed sunflower seeds in 2009 [2]. There have been multiple publications describing EV-like “plant-derived edible nanoparticles” purified from ground plant tissue for use in nutrition and therapeutics [3]. However, in the absence of known markers, it has not been possible to know whether such vesicles were truly extracellular, or simply contaminating endosomal vesicles from broken cells. A breakthrough was published in 2017 by Rutter and Innes [4] from the Indiana University Bloomington, describing the purification of EVs from intercellular wash fluids recovered from leaves of *Arabidopsis thaliana*. In this study, which employed an iodixanol density gradient purification step, multiple markers associated with intracellular endosomes were used to rule out contamination from broken cells. This protocol [5] is now enabling the plant community to purify plant EVs and characterize their contents.

Work in the Innes laboratory revealed that plant EVs are enriched in proteins associated with both biotic and abiotic stress responses [4]. At the mini-symposium, Professor Roger Innes presented ongoing work on the RNA content of plant EVs. Analyses from his laboratory have revealed that EVs contain diverse small RNA (sRNAs) species, including both small interfering RNAs (siRNAs) and microRNAs (miRNAs). Interestingly, EVs were found highly enriched in RNA molecules that are only 10–17 nucleotides in length, thus significantly shorter than canonical miRNAs and siRNAs that are typically 21 or 24 nucleotides long. These very short RNAs have been dubbed “tiny RNAs” (tyRNAs). Their short length complicates mapping the genomic origins of tyRNAs, since most reads map to multiple locations. However, when mapping those with just single matches in the genome it is clear that tyRNAs come from diverse sources and likely represent degradation products of multiple RNA classes, including mRNAs, rRNAs and PolIV-derived RNAs [6]. It is not yet clear what the function of tyRNAs may be, but their strong enrichment in EVs suggests they may indeed have a function, as it would seem wasteful to export so many nucleotides from the cell rather than recycle them. Examination of whole tissue sRNA data sets in public databases indicates that tyRNAs are more abundant in plants than mammals, but have been overlooked due to bioinformatic analysis pipelines that discard reads shorter than 18 nucleotides. Going forward, major questions that are currently under investigation are whether plant tyRNAs are taken up by pathogens via uptake of EVs [7,8], and if so, whether tyRNAs function in immunity.

Small RNAs are a class of short non-coding RNAs that mediates gene silencing in a sequence-specific manner. Professor Hailing Jin’s lab, from the University of California at Riverside, has previously demonstrated that some sRNAs from eukaryotic pathogens, such as *Botrytis cinerea*, the fungal pathogen that causes the grey mould disease on more than 1000 plant species, can transfer into host plant cells and suppress host immunity genes for successful infection [9]. To examine whether host endogenous sRNAs are delivered into fungal cells, the Jin laboratory has developed a sequential protoplast preparation protocol to isolate pure fungal cells from the infected tissue, and identified a list of host sRNAs in the purified *B. cinerea* cells. Furthermore, a drastic increase of EVs at the fungal infection sites was observed, which led them to isolate EVs from infected tissue and perform small RNA-profiling analysis. They found that the majority of the host sRNAs that transferred into fungal cells are present in the isolated EVs, suggesting that plant endogenous sRNAs are secreted by EVs and transferred into fungal cells. These sRNAs induce cross-kingdom RNA interference (RNAi) of fungal genes involved in pathogenicity. Importantly, two *Arabidopsis* EV markers, the tetraspanins TET8 and TET9 that are induced by *B. cinerea* inoculation were identified. Mutation in the corresponding EV marker genes leads to a reduced sRNA transport into fungal cells and enhanced plant susceptibility. These data suggest that exosome-borne EVs may be one of the major pathways for delivering host sRNAs into fungal cells and inducing cross-kingdom RNAi of fungal virulence genes [7].

## EVs of animal parasites in inter-organismal communication

Diverse eukaryotic parasites have been shown to export their RNA and protein molecules in EVs that are internalized by other cells [10]. The EVs serve as a mechanism for communication between parasites or between parasites and their hosts [11]. A major research focus of the EVs released from parasitic worms has been their immunomodulatory capacity; since worms release an array of immunosuppressive proteins and other molecules and some of these have been shown to have therapeutic potential in models of allergy and auto-immunity [12]. Administration of EVs released from different gastrointestinal nematodes led to suppression of inflammation in mouse models of immune pathology: *Heligmosomoides bakeri* EVs suppress the type2 innate immune response to allergens [13] and *Trichuris muris* EVs suppress inflammation in a model of colitis [14]. Several reports also suggest the EVs from these parasites are important to parasite survival, as vaccination against the EVs confers protection against infection [15,16].

Beyond the exciting therapeutic properties of worm EVs, the study of EV biogenesis and mode of action in worms provides diversity to the field of animal EV research, which is largely dominated by the study of mouse and human EVs. The parasites have co-evolved with their mammalian hosts for very long periods and understanding how worms use EVs to interact with mammals may offer fundamental insights into mechanisms of selectivity, target uptake and function. A major focus is to understand the parasite cargos that are transferred to host cells and how these cargos change host cell activity. Professor Amy Buck presented work showing that several different classes of parasite RNA are transferred in EVs to host epithelial cells. In addition to miRNAs, specific secondary siRNAs are enriched in EVs and many of these derive from novel repetitive regions in the parasite genome. These siRNAs are associated with a nematode-unique Argonaute protein that is highly expressed in, and secreted by, parasites and may serve as a key specificity determinant in which siRNAs are released from the parasite [17]. These data suggest an RNA-binding protein in the nematode mediates siRNA export in EVs and provides interesting future work on the targets of these siRNAs in host cells. The EVs in Professor Buck’s study were purified by ultracentrifugation followed by floating on a sucrose density gradient [17] and the Argonaute protein was protected from degradation by proteinase K. Quantification of the Argonaute in the EVs using a recombinant standard suggests several copies are present per EV.

Dr. Neta Regev-Rudzki from the Weizmann Institute showed how the malaria parasite, *Plasmodium falciparum*, manipulates its host immune system using EVs containing nucleic acid cargo composed of parasitic DNA and RNA.[18,19]. *P. falciparum* infected or uninfected red blood cells growth media was collected and cellular debris removed by centrifugation at 1500 r.p.m., 3000 r.p.m. and 10,000 r.p.m. The supernatant was concentrated using a Vivaflow 100,000 MWCO PES (Sartorious Stedium) and centrifuged at 150,000 x g [18]. They showed that the parasitic vesicular-DNA is released within the first 12 h post-invasion of the parasite into its host red blood cell. The DNA-harbouring vesicles are then efficiently internalized by monocytes and activate the STING pathway. They further showed that the stimulation of the immune cells is via STING-TBK1-IRF3-dependent gene induction. This study provides for the first time a rationale as to how malaria DNA gains access to host DNA sensing pathways to modulate STING signalling [18].

## EVs of bacterial pathogens in cross-kingdom interaction

EVs from prokaryotes were discovered in the 1960s and have been frequently documented in pathogenic bacteria causing diseases in humans [20–22]. Mostly referred to as outer membrane vesicles (OMVs) or membrane vesicles (MVs), their biochemical properties, molecular components and pathways of vesicle release from donor cells and uptake into recipient cells have been defined [23]. In the context of cross-kingdom communication, several studies discovered both immunogenic and immune-suppressive activities of bacterial EVs in human cells [23]. EVs from plant-interacting bacteria were first described in the 1980s from cultures of the plant-interacting bacteria *Erwinia amylovora* and *E. carotovora* [24,25]. Later, EVs from *Xanthomonas campestris* pv. *vesicatoria* were also found during the infection process in pepper [26]. Yet, our knowledge of EVs from plant-interacting bacteria and their role in communication with plant cells is still rudimentary. Initial studies from *Pseudomonas, Xanthomonas* and *Xylella* species revealed both immunogenic activities and immune-suppressive capacities as indicated from vesicle proteome analysis [26–29]. EVs can promote infection success additionally by regulating bacterial-to-plant cell attachment [30]. Outstanding questions are how EVs affect the plant’s immune response, what is their cargo and what are the plant targets. Dr Silke Robatzek, from the LMU of Munich, discussed exciting new observations made with EVs from *Pseudomonas syringae* pv. *tomato* (*Pto*). Although immunogenic when initially eliciting plants, EVs from *Pto* have the capacity to suppress pattern-triggered immunity. Consistently, *in planta* expression of selected EV cargo impaired prototypic microbe-associated molecular pattern (MAMP)-induced immunity, suggesting that these bacterial proteins can target components of the plant immune system. Her current hypothesis is that *Pto* releases EVs that interact with plant cells such that EV cargo could be translocated into host cells, an interesting question to be addressed in future research.

EVs were purified from culture-grown *Pto* using a combination of filtration, ultra- and density step-gradient centrifugation and visualized with transmission electron microscopy (TEM), dynamic light scattering (DLS) and nanoparticle tracking analysis (NTA). Briefly, the supernatant of a 500 ml bacterial culture was filtered, and then ultra-centrifugated at 100,000 x g. The resuspended pellet was loaded onto a sucrose density gradient and ultra-centrifugated at 160,000 x g. Fractions were recovered and again ultra-centrifugated at 100,000 x g. Final pellets were resuspended and analysed by TEM, DLS and NTA.

## EVs of fungal pathogens in cross-kingdom interaction

It has been shown previously that pathogenic fungi release EVs and they are used in cross-kingdom modulation of host cells. *Cryptococcus gattii*, an encapsulated yeast-like fungal pathogen of humans and other animals, releases EVs and similarly to its sibling species *C. neoformans*, uses them in inter-organismal communication. Studies presented by Dr Ewa Bielska from the University of Birmingham, UK, showed that EVs released by a deadly isolate from the Pacific Northwest outbreak of cryptococcosis are able to increase virulence of less pathogenic *C. gattii* isolates *in vitro* [31]. Fungal proteins and RNAs associated with these EVs can overtake and modulate fungal cells inside mammalian phagocytes, allowing them higher internal proliferation in macrophages. This phenomenon may be associated with the presence of heat shock proteins and pyruvate kinase inside *C. gattii* EVs, where both classes of proteins are protective against heat stress in fungi [32]. Indeed, fungal growth at 37°C can be further accelerated in the presence of the EVs. Pre-treatment of macrophages with the EVs also leads to the small but significant increase of the intracellular proliferation of the less pathogenic *C. gattii* isolate [31], which may be associated with reduced interferon γ levels release by white blood cells. Contradictory to studies performed in *C. neoformans* [33], EVs isolated from *C. gattii* do not enhance phagocytic activity of macrophages. Incorporation of the fungal EVs by mammalian white blood cells is based on several endocytic routes and can be blocked using inhibitors of endocytosis like latrunculin A, cytochalasin D or methyl-*β*-cyclodextrin. *In vitro* studies showed that the uptake of fungal EVs by macrophages might be very fast with a half time to peak internalization of 17.5 min and that within initial 15 min EVs can colocalize with phagocytosed yeasts in the phagosome [31].

Isolation of EVs from the cryptococcal cells was performed using classical differential centrifugation followed by ultracentrifugation method (100,000 x g, 1 h at 4°C; [31]). Analysis of protein concentration was performed using Micro BCA Protein Assay Kit (Thermo Fisher Scientific #23235) [31]. To visualize EVs microscopically during interaction with a host, a fluorescent lipophilic dye Vybrant DiI can be used. Additional washing steps are required to remove excess dye, but this leads to a reduction of the EV yield.

While fungal EVs have been reported several times in human-associated and pathogenic species, information on EVs from plant pathogenic fungi remains scarce [34]. *Botrytis cinerea* is a broad range fungal plant pathogen that causes the grey mould disease in many crop species. During host infection, *B. cinerea* delivers siRNAs into host plant cells that hijack the plant RNAi pathway to silence plant innate immunity [9], a communication channel that is probably also employed by other types of plant-interacting microbes [35]. Exchange of siRNAs between *B. cinerea* and its host plants is bi-directional [36], as the delivery of plant-derived siRNAs into *B. cinerea* occurs via EVs [7]. However, whether fungal plant pathogens deliver biomolecules including siRNA effectors also via EVs into plant cells is still to be discovered. The group of Dr Arne Weiberg at the LMU, Munich, presented data on the isolation of EV-like structures from the culture supernatant of *B. cinerea* by stepwise filtration (0.22 μm) and ultra-centrifugation at 120 min at 100,000 x g. *B. cinerea* EV-like structures were observed by TEM, DLS and NTA resembling EV shape and size range as previously found in animal and plant species. This EV-like fraction contains *Botrytis* siRNAs that target plant host immunity genes [8]. Treatment of EV-like particles with RNA nuclease A was unable to eliminate *Botrytis* siRNAs indicating their protection by encapsulation within the EV-like particles. To further consolidate EV research in *Botrytis* and other fungal plant pathogens, fulfilment of the minimal information for studies of EVs is required. Moreover, studies to explore the biological functions of EVs from *Botrytis* and other fungal plant pathogens including delivery of siRNA effectors into host plants are necessary.

## EVs in oomycete effector delivery

Filamentous plant pathogens include the fungi, described above, and oomycetes, which are related to diatoms and brown algae. Oomycetes, such as the infamous late blight pathogen *Phytophthora infestans* [37], cause a wide range of devastating diseases of economic and environmental importance [38]. *P. infestans* secretes a range of virulence determinants called effectors that can act either outside (apoplastic) or within (cytoplasmic) the plant cell, several of which have been shown to suppress host immunity [39]. Amongst the cytoplasmic effectors are the RXLR class, so-called for the conserved amino acid motif Arg-any amino acid-Leu-Arg, which is required for these effectors to be translocated into plant cells [40]. Recently, delivery of two RXLR effectors has been visualized from finger-like infection structures called haustoria, which form intimate associations with plant cells, to their sites of action in host nuclei [41,42]. The precise means by which RXLR effectors are secreted from *Phytophthora* species is poorly understood [41].

The group of Professor Paul Birch at Dundee University presented preliminary data on the isolation of EV-like structures from the culture filtrate (CF) of *P. infestans* using filtration and ultracentrifugation. After growth for 48 h in liquid medium, mycelium was removed and CF was centrifuged successively at 2000 x g for 10 min, 10,000 x g for 30 min, and 40,000 x g for 60 min at 4°C [4]. To determine if EV-like structures were present in the CF, the pellet (P) fractions were examined after ultracentrifugation using TEM.  Potential double lipid-layered EV-like structures (~100 nm in diameter) were presented in P fractions. Preliminary proteomic analyses of the P fractions indicated a range of proteins, such as Rabs, motor proteins, annexins and heat-shock proteins, which have previously been associated with EVs. In contrast, proteins predicted to be conventionally secreted, such as some apoplastic effectors SCR108, SCR122, EPIC1 and EPIC4 and cell wall degrading enzymes [42] were specifically enriched in the supernatant samples. Critically, certain RXLR candidate effectors were detected only in pellet samples, providing compelling evidence that these effectors are delivered using EVs. Future work will aim to determine whether indeed RXLR effectors are secreted and translocated by means of EV-like structures; and how such effectors become associated with EVs during their biogenesis.

## Analysing EV small RNAs in inter-organismal interactions

All types of EVs examined so far contain a variety of sRNAs suggesting that these are key “messages” of inter-organismal communication. Many questions are currently being addressed regarding EV sRNAs: How are specific sRNAs selected for loading into EVs? Are particular EV sRNAs selectively received by defined host cells? What are the functions of the received sRNAs within the host cell? The group of Dr Cei Abreu-Goodger from Langebio, Mexico, focuses on bioinformatic challenges when analysing and interpreting high-throughput sRNA sequencing data coming from experiments of parasite EVs within infected host tissues. In these experiments, the first step is to separate the parasitic from the host sRNAs, yet an important fraction of small sequences can map perfectly to both genomes. The number of co-mapping reads depends on the size and nucleotide composition of the reads, the sizes of the reference genomes, the phylogenetic relationship between the reference organisms, and the genomic region a sRNA read is derived from; for instance, conserved miRNAs and ribosomal RNA reads have high chances to co-occur in both reference genomes [43]. Most bioinformatic tools have been designed to ignore such “multi-mapping” reads by default, and most pipelines discard reads that map multiple times or equally well to two reference genomes, causing particular loss of sequences which have high potential to function in cross-species communication. Isolating EVs from pure parasitic or microbial cultures for sRNA sequencing can help to create a species-specific sequence dataset to validate the origin of ambiguous sRNA reads in interaction studies. Experiments designed for differential expression analysis, e.g. comparing infected and control samples, can also help to locate the foreign sRNAs. On the bioinformatic level, multi-mapping reads can be partially disambiguated by preferentially mapping to the genomic region that has the most uniquely mapping reads [44]. Further, different types of short sRNA assembly can extend reads by a few bases, which lead to more accurate placement of sRNAs [43]. The best results are achieved by combining appropriate assembly and mapping bioinformatic strategies, with experiments that allow for differential expression analyses [43]. The next challenge for bioinformatics will be to predict targets for sRNAs involved in inter-organismal communication, especially for those that are bound to non-canonical Argonaute proteins [17].

## Meeting the MISEV compliance goals in inter-organismal EV research

The scientific interest in the functions of EVs in cross-kingdom and inter-organismal communications is growing rapidly [1,45,46], in particular driven by a general discussion if there is an EV transfer and effective uptake of RNA by the opposite specimen [47–51]. Due to different scientific questions, the EV science community is using a multitude of experimental setups and biological systems, isolation techniques and instrumentations to characterize EVs and their subpopulations. These creates a vast heterogeneity of published methods, applied protocols and makes the classification of EVs and the scientific evaluation of the derived results very laborious and unequal. This heterogeneity in numerous specimens and applications motivated the EV community to develop international guidelines and general recommendations. The final goal is to standardize EV nomenclature, applied methods, working protocols, and how to report results in an understandable and unmistakable way. The first paper supporting these objectives, the “Minimal Information for Studies of EVs” (MISEV guidelines) was published in 2014 [52], further discussed in 2017 [53], and a new updated 2018 version has been published recently [54]. To maintain this initiative, a further review was published, which focuses mainly on the methodological guidelines [55]. This initiative was further supported by the “EV Track” consortium [56], which asks for more transparent reporting to facilitate interpretation and replication of EV experiments. To achieve this, a crowdsourcing knowledge database was developed (http://evtrack.org) that centralizes EV biology, markers and methodology. With “EV Track”, the final objective is to stimulate authors, reviewers, editors and funders to put experimental guidelines into valid and reproducible laboratory practice. In this context, the most prominent vesicle and biomarker databases are EV Track [56], ExoCarta [57], now called Vesiclepedia [58], and EVpedia [59,60]. However, the latest MISEV version [54] and the data presented in “EV Track” and other databases have still the major scientific focus on human samples, human cell culture models, human liquid biopsy and lab animals like mouse and rat. Most researched scenarios of inter-species transfer are so far host-pathogen, host-parasite and host-microbiome interactions [10,45]. As we encourage, EV research in inter-species and cross-kingdom communications that includes animals, plants, fungi and other unicellular microorganism, Professor Michael W. Pfaffl from the Technical University of Munich in Weihenstephan drew in his talk a clear perspective on the next goals to succeed. Primarily, the community needs better repositories for reliable molecular and phenotypic markers, with focus on non-vertebrate species. These databases should store various type of molecular biomarker information, e.g. nucleic acids (RNA, DNA, various small-RNA, microRNA, long non-coding RNA, and circular RNA), proteins (surface, intracellular, organelle, and secreted proteins), EV cell wall components (lipids, carbohydrates, glycolipids, and other polymers) and metabolomic markers characteristic from the descent cell. Further phenotypic EV characteristics can be stored as well, e.g. size range, shape, density, granularity, viscosity, zeta-potential, containing vacuoles, self-fluorescence and the general stability of EVs. In conclusion, such updated repositories and guidelines will support the general knowledge of inter-organismal EV biology and  will allow precise classification to which organism an EV belongs, and what is the tissue of origin. It will enable to trace the EVs during biogenesis, transfer, and in the recipient organism and/or target tissue. These new established standards and updated knowledgebase would increase the validity of EV cross-kingdom research. Therefore, the already existing databases and guidelines need to be enlarged and completed by:

- new biological species, which are involved in inter-organismal communication;
- the connected EV isolation methods due to differences in overall cell architecture and cell wall compositions, e.g. from pathogens, helminths or microorganisms;
- new vesicle types and characteristics based on vesicle architecture or way of release and biogenesis;
- new surface marker proteins for isolation and identification, e.g. by antibody and/or bead-based systems, by fluorescence-based nanoparticle tracking analysis or flow cytometry;
- new intracellular markers, e.g. new protein, micro-RNA or small-RNA biomarker signatures;
- new phenotypic markers, like size, shape, density or other significant visible characteristics;
